# Video Consultations and Safety App Targeting Pregnant Women Exposed to Intimate Partner Violence in Denmark and Spain: Nested Cohort Intervention Study (STOP Study)

**DOI:** 10.2196/38563

**Published:** 2023-03-20

**Authors:** Karen Andreasen, Antonella Ludmila Zapata-Calvente, Stella Martín-de-las-Heras, Aurora Bueno-Cavanillas, Berit Schei, Sarah Dokkedahl, Sabina de León de León, Rodrigo Fernandez Lopez, Alba Oviedo-Gutiérrez, Lea Bo Sønderlund Ankerstjerne, Jesús L Megías, Khalid Saeed Khan, Vibeke Rasch, Ditte S Linde

**Affiliations:** 1 Department of Clinical Research University of Southern Denmark Odense Denmark; 2 Department of Gyneacology and Obstetrics Odense University Hospital Odense Denmark; 3 Open Patient Data Explorative Network Odense University Hospital Odense Denmark; 4 Brain and Behavior Research Center University of Granada Granada Spain; 5 Biomedical Research Institute Department of Forensic Medicine University of Malaga Malaga Spain; 6 Department of Preventive Medicine and Public Health University of Granada Granada Spain; 7 Center for Biomedical Research in Epidemiology and Public Health Network-Spain University of Granada Granada Spain; 8 Institute of Public Health Norwegian University of Science and Technology Trondheim Norway; 9 Department of Obstetrics and Gynaecology Sct. Olavs University Hospital Trondheim Norway; 10 Department of Psychology University of Southern Denmark Odense Denmark

**Keywords:** intimate partner violence, gender-based violence, domestic violence, abuse, telemedicine, video counseling, app, acceptance, safety, feasibility, empowerment

## Abstract

**Background:**

Intimate partner violence (IPV) during pregnancy is a public health issue with wide-ranging consequences for both the mother and fetus, and interventions are needed. Therefore, the Stop Intimate Partner Violence in Pregnancy (STOP) cohort was established with the overall aim to identify pregnant women exposed to IPV through digital screening and offer women screening positive for IPV a digital supportive intervention.

**Objective:**

The aim of this study was to (1) introduce the design and profile of the STOP cohort study, (2) assess the feasibility of implementing digital IPV screening among pregnant women, and (3) assess the feasibility of implementing a digital supportive intervention targeting pregnant women exposed to IPV.

**Methods:**

Pregnant women attending antenatal care in the Region of Southern Denmark and in Andalucía, Spain were offered digital screening for IPV using validated scales (Abuse Assessment Screen and Women Abuse Screening Tool). Women who screened positive were eligible to receive a digital supportive intervention. The intervention consisted of 3-6 video consultations with an IPV counselor and a safety planning app. In Denmark, IPV counselors were antenatal care midwives trained by a psychologist specialized in IPV, whereas in Spain, the counselor was a psychologist.

**Results:**

Data collection started in February 2021 and was completed in October 2022. Across Denmark and Spain, a total of 19,442 pregnant women were invited for IPV screening and 16,068 women (82.65%) completed the screening. More women in Spain screened positive for exposure to IPV (350/2055, 17.03%) than in Denmark (1195/14,013, 8.53%). Among the women who screened positive, only 31.39% (485/1545) were eligible to receive the intervention with only 104 (21.4%) of these women ultimately receiving it.

**Conclusions:**

Digital screening for IPV among pregnant women is feasible in an antenatal care context in Denmark and Spain; however, a digital supportive intervention during pregnancy appears to have limited feasibility as only a minor subgroup of women who screened positive for eligibility received the intervention. More research is needed on how to best support pregnant women exposed to IPV if universal IPV screening is to be implemented in antenatal care.

## Introduction

### Background

Intimate partner violence (IPV) is a major public health issue. Pregnant women are particularly vulnerable to IPV, as the violence affects both the women and their unborn infants. Violence during pregnancy has been reported to increase the risk of a broad range of disorders, including pregnancy-related complications and depression as well as adverse perinatal outcomes such as preterm birth, low birth weight, perinatal death, and shortened breastfeeding duration [1-11]. The World Health Organization defines IPV as “a behavior by an intimate partner or ex-partner that causes physical, sexual or psychological harm, including physical aggression, sexual coercion, psychological abuse and controlling behaviors” [12]. The estimated prevalence of IPV exposure during pregnancy varies; however, a meta-analysis of 152 studies from more than 50 countries showed that the average prevalence was 9.3% for physical violence alone, 5.5% for sexual abuse, and 18.7% for emotional abuse [13]. Additionally, the COVID-19 pandemic lockdowns and subsequent social isolation have increased the overall prevalence of IPV worldwide [14].

Antenatal care is considered a window of opportunity to reduce IPV in general, because this is a time when women are in regular contact with health care providers and pregnant women’s concern for their unborn child may motivate them to share their exposure to violence [15,16]. When pregnant, the vast majority of women attend a screening program aimed at assessing a variety of risks; although IPV is already included among these screened risks in some countries, this is not currently the case in Denmark and Spain. Nevertheless, studies have shown that women may not disclose IPV during face-to-face antenatal consultations due to self-blame or presence of their violent partner [15,17]. One way to address this issue would be to offer a digital self-administered screening tool for pregnant women [17-20], and if screening positive, to subsequently offer an intervention [21,22].

Previous studies on digital interventions have primarily focused on the effect of screening in combination with access to a safety app, showing conflicting results as to the potential effects on reduction of IPV and related health outcomes. However, a 2019 randomized controlled trial (RCT) from the United States showed that pregnant women found an educative computer intervention combined with a booster session with a health care provider to be helpful and that it had potential to reduce IPV [20]. Further, a recent study from Norway investigating the effect of a digital intervention for the prevention of IPV indicated that women found the antenatal care setting a safe place both to respond to the IPV questions and to watch a video with safety behaviors. Yet, the participating women recommended that the digital intervention should be supplemented with a supporting dialogue with a midwife [19]. Hence, a trusting relationship with a supportive health care provider may be of importance for the ongoing dialogue about IPV when delivering digital screening and interventions. To the best of our knowledge, no other studies have implemented a digital intervention (combining screening and live counseling sessions with a health care professional) toward establishing a trusting relationship over a longer period of time. The “Stop Intimate Partner Violence in Pregnancy (STOP)” project was designed to accommodate this.

The overall aim of the STOP cohort study was to implement digital screening for IPV in antenatal care, as well as to develop and evaluate the effect of a digital supportive intervention for pregnant women exposed to IPV. The objectives of the STOP study were to (1) assess the feasibility of implementing digital IPV screening among pregnant women in antenatal care in Denmark and Spain; (2) assess the feasibility of implementing a digital intervention in the form of video counseling and a safety app targeting pregnant women exposed to IPV; (3) estimate the prevalence of different types of IPV according to different screening tools; (4) explore in-depth the feasibility and acceptability of a supportive digital intervention among the women who received the intervention and the health care personnel conducting the counseling; (5) evaluate whether the intervention could reduce the severity of IPV, postpartum depression, and improve empowerment and safety actions among pregnant women exposed to IPV; and (6) assess the significance of the timing of video counseling during pregnancy through a pilot RCT.

### Study Objectives

Among the general STOP study goals stated above, the aim of this study was to (1) describe the design and profile of the cohort study, (2) assess the feasibility of implementing digital IPV screening among pregnant women, and (3) assess the feasibility of implementing a digital intervention in the form of video counseling and a safety planning app targeting pregnant women exposed to IPV. The results of the remaining objectives will be addressed in future publications. Within the concept of feasibility, we refer to the focus area of “implementation,” which is defined as “the extent…in which an intervention can be fully implemented as planned and proposed” [23]. For this context, we interpreted implementation as whether pregnant women participate in the digital screening when offered and whether pregnant women exposed to IPV participate in the digital intervention.

## Methods

### Study Setting and Context

The STOP study is an international collaboration study between the Region of Southern Denmark and the University of Granada, Spain. The Region of Southern Denmark has a population of 1.2 million, which accounts for one-fifth of the total Danish population. In the Region of Southern Denmark, antenatal care is offered at four hospitals: Lillebaelt Hospital, Odense University Hospital, South Jutland Hospital, and Southwest Jutland Hospital. In Spain, the study was conducted in the region of Andalusía, which has a population of 8.5 million across eight provinces. The study took place at 42 routine public antenatal care centers in the provinces of Granada, Jaén, Málaga, and Córdoba. Antenatal care is part of both the Spanish and Danish public health systems and is offered freely for all pregnant women.

### Study Design

#### Implementation of Digital Screening in Antenatal Care

In Denmark, the screening for IPV was incorporated into the Patient Reported Outcome (PRO)-data system, which is a digital questionnaire offered to all pregnant women in the first trimester. PRO-data in pregnancy focuses on general health conditions, including screening questions for IPV. The pregnant women received the questionnaire through the app “My Hospital,” which is used at all hospitals in the region. Once they completed the questionnaire, a summary of their responses was attached to their electronic health record. The answers were discussed with a midwife at the first antenatal care appointment, which is scheduled at approximately gestational week 16.

In Spain, pregnant women were invited to fill in a digital IPV questionnaire using a specially designed app for the STOP project during their first routine antenatal consultation in their first trimester. They were asked to respond to IPV screening questions and to provide various sociodemographic characteristics. The app prompted the women who screened positive for IPV to provide their contact information (telephone number and/or email address) if they wanted to participate in the digital intervention and to be contacted by a psychologist. STOP aimed to screen 11,000 and 2000 women in Denmark and Spain, respectively.

#### Screening Tools for IPV

The screening was based on validated psychometric tools, specifically the Abuse Assessment Screen (AAS) and the Women Abuse Screening Tool (WAST). Through a 5-item instrument with binary response options, AAS measures physical, emotional, and sexual violence from a partner or close relative within or prior to the past 12 months [24]. The WAST measures conflict and tension with the partner through a 2-item instrument that rates tension/conflicts on a 3-point scale ranging from 0 to 2 [25]. Women were considered to be exposed to IPV (screened as IPV-positive) if they reported violence on either the WAST or AAS. The cut-off score for the WAST was a positive response to the extreme categories (score of 2) for either item or a positive response to the middle categories (score of 1) for *both* items. The cut-off score for AAS was a positive answer to any type of violence perpetrated by the current partner or ex-partner regardless of the timing. If women screened positive in either the AAS or WAST, they were asked to rate the severity of violence through the Index of Spouse Abuse (ISA) scale. ISA is a 30-item instrument designed to measure the severity of physical and nonphysical violence [26].

#### Inclusion and Exclusion Criteria for the Digital Intervention

Women were considered eligible for the digital intervention if they reported severe violence/tensions with a partner on the WAST (score 2); in Spain, a positive response to the middle categories (score of 1) in *both* items also triggered eligibility. In Spain, all women with any positive AAS screen were considered eligible. In Denmark, AAS-based eligibility was dependent on several factors: either a positive response for any time within the past 12 months or an existing fear of a partner/ex-partner ever; after 11 months, the Danish criteria were changed to align with the Spanish criteria. In both countries, women were excluded from the intervention if they (1) could not be informed about the study without their partners or other family members knowing about it, (2) did not have the mental or physical capacity to participate in the study, (3) did not understand either Spanish or Danish, (4) lacked internet access and/or smartphone devices, or (5) were under the age of 18 years in Denmark and 16 years Spain. Pregnant women were eligible for inclusion until gestational week 12 in Spain and until gestational week 25 in Denmark.

#### Pilot Phase of the Digital Intervention

The first weeks of the project were used as a pilot phase, where different inclusion criteria were tested. In Denmark, this phase lasted for 4 months in which women were only offered the intervention if they reported a certain severity of violence on the ISA scale. Upon evaluation of the pilot phase, it was decided to also offer the intervention to women who reported exposure to IPV according to the WAST and AAS. Further, the Danish AAS eligibility criteria were changed after 11 months, as mentioned above. In Spain, the screening process was conducted as a pilot for the first 2 weeks. During this phase, women who screened positive were also eligible for the video counseling intervention if they reported *any* of the middle categories (score of 1) of “tension” or “difficulty” with their partner in the WAST. Once the pilot was evaluated, the WAST cutoff was changed, and women were eligible only if they reported *both* middle categories or any of the extreme scores.

Despite being a pilot period, the women in both countries who screened positive during this phase were included in the project. All pilot data are included in the study.

#### Recruitment for the Digital Intervention

In Denmark, a project counselor contacted the women by phone and informed them briefly about the study. If a woman was deemed to be eligible and indicated interest in participating in the study, they were invited to participate in the study at their first antenatal care visit (~gestational week 16). Danish legislation requires that women must accept to be contacted for research; hence, women could not be invited into the intervention study until they had given their consent to this at their first antenatal care appointment. The inclusion period was extended until after the second antenatal care appointment (~gestational week 22). The reason for this was to allow for the option to include women who attended their first antenatal care appointment with their partner, but who came to their second appointment alone. If a potential participant was unreachable, a project counselor tried to establish contact by phone or email at 3 different occasions within 6 weeks. If this was not possible, the woman was considered ineligible for the study. In Spain, women gave informed consent to participate in the screening before answering the digital questionnaire at their first antenatal care appointment. If a woman screened positive, they were contacted within the following days and invited to participate in the study. When the psychologist contacted the woman, a second informed consent was audio-recorded if they accepted to participate in the study. A project counselor tried to reach potential participants up to 3 times by phone call and twice via email within a 3-day interval. If women could not be reached within this period, they were considered ineligible for the study.

#### Cocreation of the Digital Intervention

The intervention was cocreated with women who previously had been exposed to IPV (n=6), psychologists, midwives, and nongovernmental organizations (NGOs) working within the field of IPV (n=13). Focus group discussions, individual interviews, and workshops were conducted in both Denmark and Spain to discuss and receive input about the needs of women exposed to IPV, their ways of handling IPV, and their expectations of a digital intervention. The Spanish cocreation process has been published [27]. Based on the input given by the women and health providers/NGOs, the following themes for an intervention were identified: (1) lack of acknowledgement of being exposed to IPV, (2) ambivalent emotions toward the partner, (3) lack of resources and worries about being on their own, (4) low self-esteem making it difficult to make decisions, and (5) isolation. These themes were incorporated into the design of the digital intervention, and translated into content and technical specifications for the video counseling and the safety planning app in line with elements from Mary Ann Dutton’s Empowerment Model [28] and the Psychosocial Readiness Model [29]. Specifically, elements concerning safety planning, enhancement of decision-making and problem-solving, and healing psychological reactions of the abuse were included [28], as well as elements that strengthen internal factors such as awareness, perceived support, and self-efficacy, which affect a woman’s likelihood of movement toward change [29].

#### Content of the Digital Intervention

The digital intervention consisted of video consultations with an IPV counselor as well as access to a safety planning app.

All women were initially offered six video consultations unless the IPV counselor and the woman jointly agreed during the intervention period that fewer sessions were needed. The sessions specifically addressed the following topics: (1) evaluation of abusive behaviors; (2) safety planning, network, and resources; (3) psychoeducation; (4) self-esteem and self-care; and (5, 6) empowerment, choice-making, and problem-solving. The consultations were scheduled every other week at a time where the women felt comfortable. In Denmark, the My Hospital app was used to organize and host the video consultations, whereas the Linkello Medical platform was used in Spain. In Denmark, the IPV counselors were midwives who were trained by a psychologist specialized in IPV. In Spain, a psychologist experienced in IPV counseling conducted the counseling.

**Figure 1:**
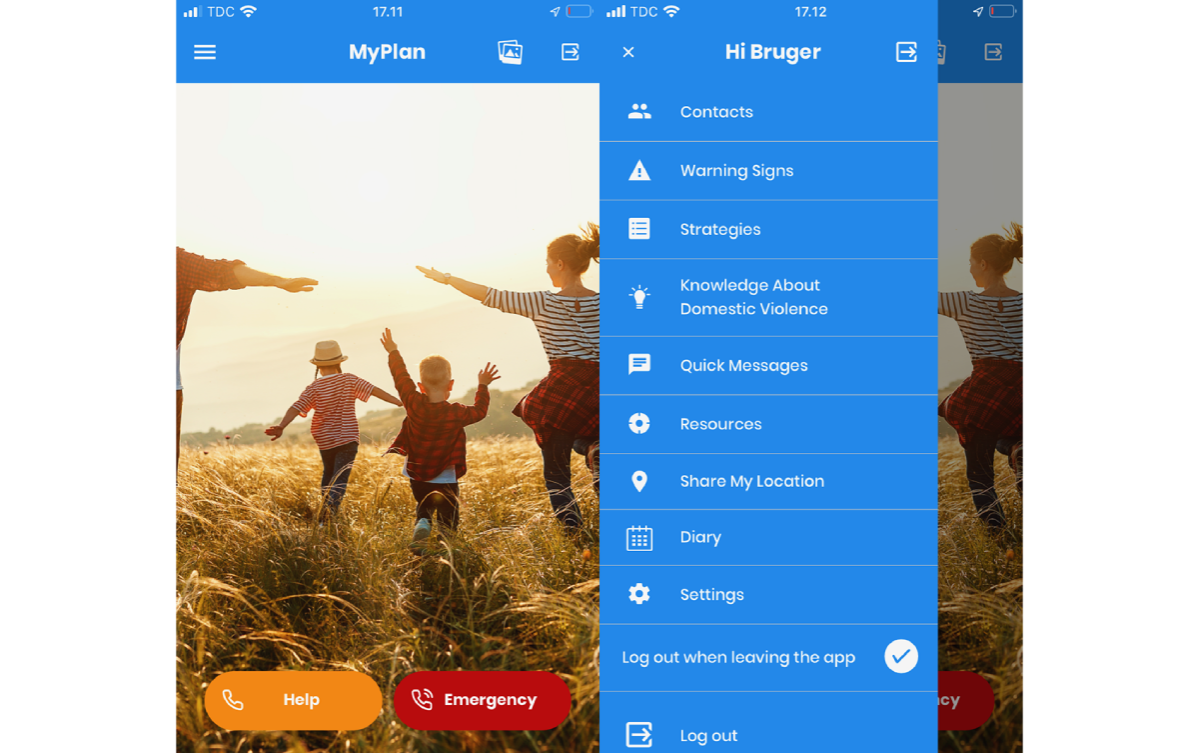
Screenshot of the MYPLAN safety app.

In both Denmark and Spain, an adapted version of the smartphone safety planning app “MYPLAN” was used. The MYPLAN app was developed by Glass et al [30] and is freely available. A safety plan is a personal and practical plan designed by a woman exposed to violence to minimize their risk of danger and exposure to violence. By digitalizing the safety plan into an app, it is easily accessible for women to help remind them of their own strategies and resources. The safety planning element was hidden in a pregnancy app, and for security reasons, the women had to log on to the pregnancy app to access their safety plan. Once a woman had logged on, the front page consisted of two buttons: “Help” and “Emergency” (Figure 1). Both buttons consisted of default contact information for relevant resources that could help the woman in case they needed help or were in an active emergency. The woman could edit the information to fit their needs. The menu page provided an overview of other features in the app (Figure 1). The “Contact” feature contained a more detailed list of different local and national organizations that support women exposed to violence. In the “Warning signs” feature, the woman could add signs that “triggered” the violence or specific situations where the violence occurred. Under “Strategies,” they could note down coping strategies, while “Knowledge About Domestic Violence” provided information and links to other resources relevant for women exposed to violence. Under the “Quick Messages” feature, a woman could enable a predetermined message to emergency contacts while also having the possibility to note matters of importance in a “Diary” feature. In case the woman needed to exit the app quickly, they could press the “quick-exit” button, and return to the camouflaged part of the app.

Before the study commenced, IPV counselors participated in a 3-day training course focusing on the theoretical framework of the video counseling sessions. The training also covered communication techniques, the use of the safety planning app, and best practices when conducting video counseling. In line with Dutton’s empowerment model [28], which describes the importance of therapist self-care and the need for the counselors to be involved in an emotionally supportive environment and in routine self-care activities outside the counseling context, all counselors were supervised monthly by a senior psychologist. Additionally, throughout the study period, meetings were scheduled between study partners and the IPV counselors to discuss and monitor the intervention and screening.

#### Pilot RCT

A pilot RCT (ClinicalTrials.gov registration NCT04978064) was nested within the cohort study using Zelen’s [31] design. Twenty women from Spain and Denmark were individually randomized to either an intervention or control group, where the control group received the intervention with an 8-week delay.

### Outcome Measures

#### Quantitative Outcomes

The outcomes of interest for the digital intervention were (1) reduction of the severity of IPV, (2) empowerment, (3) safety planning, (4) pre/postnatal depression, and (5) acceptability and (6) feasibility of the intervention.

Data on the severity of IPV, empowerment, safety planning, and depression were collected pre- and postintervention through questionnaires at study inclusion and 1 month after the intervention had ended. Change in severity of exposure to IPV was assessed through the ISA tool. Level of empowerment was assessed through the Measure of Victim Empowerment Related to Safety (MOVERS), a validated 13-item survey with questions within three domains of empowerment [32]. Further, signs on pre-/postnatal depression were measured through the Edinburgh Postnatal Depression Scale (EPDS), which is a 10-item validated questionnaire aimed at detecting postnatal depression [33]. The women’s ability to carry out safety behavior actions was measured through a revised version of the 22-item Safety Action Checklist, where the women specified whether they had used different safety actions and how helpful these actions had been [34].

In Denmark, sociodemographic characteristics for the women included in the intervention study were retrieved from the patient records. Permission to do this was obtained directly from the woman and from the National Data Protection Agencies. In Spain, sociodemographic characteristics were collected through a digital questionnaire during the screening.

#### Qualitative Outcomes

In-depth knowledge of acceptability and feasibility of the intervention were explored through semistructured individual interviews with a subgroup of the participants after the intervention had finished and with all IPV counselors. An interview guide was developed based on the Model for Assessment of Telemedicine (MAST) applications [35]. Informed consent for the interviews was obtained and data collection stopped once data were saturated. All interviews were audio-recorded and later transcribed.

### Data Management

In both countries, study data were entered directly to secure web-based databases. In Denmark, Research Electronic Data Capture (REDCap) was used, which is a secure, web-based software platform hosted at Region of Southern Denmark. In Spain, Heroku was used, and data were stored in a secure OneDrive server hosted by the University of Granada. Heroku is a cloud-based platform for creating, hosting, and managing digital apps.

### Ethical Considerations

For safety reasons, women were only eligible to receive the intervention if they attended antenatal care without their abusive partner or otherwise could be contacted without their partner knowing. All IPV counselors and midwives followed the hospital guidelines regarding referral due to abuse. In Denmark, the national guidelines on pregnancy instruct all health care providers to routinely ask pregnant women about their relationship and well-being to identify individual need for help [36], and if it is concluded that the woman needs additional support or help, this is initiated either through social services, additional visits by home visiting nurses, or the antenatal family clinic, which provides specialized antenatal care for vulnerable women. In Spain, health care providers are advised to inform the social services if suspecting a woman is subjected to abuse. However, none of the Spanish midwives had access to the women’s survey answers; only the IPV counselors could access this information. If the counselors identified severe or life-threatening abuse, the principal investigator would be notified, and the woman would be treated according to the standard Spanish protocol.

Eligible women received written information about the project and all women were asked to give their informed consent (verbally or written) upon inclusion. The video consultations were flexible as to where and when they took place to ensure the women felt safe during the consultations. If the video counseling was interrupted by an abusive partner, the counselor would change the conversation topic to pregnancy-related health problems. Further, the video sessions were conducted through a safe technical setup, and the app was disguised in the previously described pregnancy app that required login and had a “quick-exit” button.

In Denmark, the STOP study was assessed by the Regional Committee on Health Research Ethics for Southern Denmark, and according to the Danish regulation it did not require ethical clearance (ethical review number S-20200129). In Spain, the Andalusian Research Ethics Committee approved the project, including the screening tool, counseling intervention, and the pilot RCT (ethical approval numbers 202072112495, 2021128101651, and 202167133116).

## Results

### Participant Screening and Engagement

**Figure 2:**
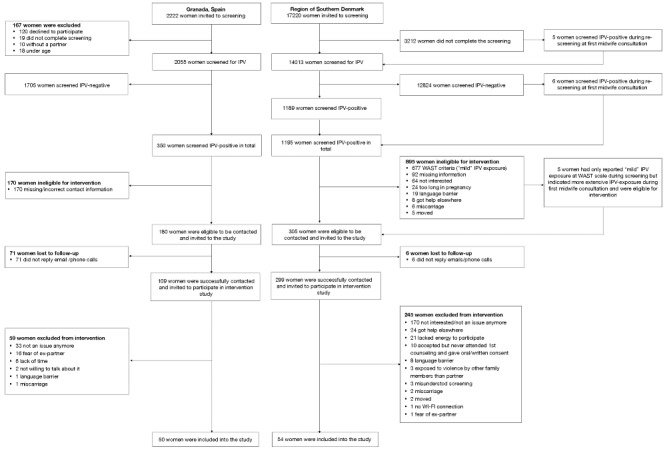
Flowchart of the STOP study. IPV: intimate partner violence; WAST: Women Abuse Screening Tool.

Data collection started in February 2021 and finished in October 2022. Over the study period, a total of 19,442 pregnant women were invited for IPV screening across Denmark (n=17,220) and Spain (n=2222), with the vast majority residing in Denmark (88.57%) (Figure 2), which was in accordance with the study protocol. Overall, 16,068 (82.65%) women completed the screening, with 81.38% (14,013/17,220) in Denmark and 92.48% (2055/2222) in Spain. A total of 1545 of the 16,068 women (9.62%) screened positive for IPV exposure, with more women screening positive in Spain (350/2055, 17.03%) than in Denmark (1195/14,013, 8.53%).

Of the 1545 women who screened IPV-positive, 485 (31.39%) were eligible for the intervention and 104 women (21.4%) were enrolled into the study, including 50 Spanish women and 54 Danish women. In Denmark, the main reason for exclusion was that women reported minor IPV exposure (eg, “some tension” or “some difficulty”) with their partner on the WAST, and despite being IPV-positive they were ineligible for inclusion there, whereas the main reason in Spain was omitting to provide contact information. Among the women who met the inclusion criteria, 408 were successfully contacted, whereas 77 women were lost to follow-up, most of whom resided in Spain (n=71, 92%). Among the 408 women contacted, 304 (74.5%) declined to participate in the intervention study; many stated that IPV was no longer an issue and others indicated that they were not interested in participating (Figure 2). Additional reasons were that the women feared their partner/ex-partner, got help elsewhere, lacked energy to participate, or that they never attended the sessions after initially accepting to participate.

### Participant Characteristics

Sociodemographic and antenatal characteristics of the women included in the intervention study are summarized in Table 1. In Denmark, the mean age among participants was 28.7 years and the median time of inclusion in the study was in gestational week 22. In Spain, the mean age was 31.6 years and the median time of inclusion in the study was in gestational week 10. In Spain, most women were expecting their second child, whereas most of the participants in Denmark were expecting their first child. In both countries, the majority indicated to both having a partner and living with a partner. Further, most women had a university/college degree in both Denmark and Spain, were employed, and had a person they trusted when they faced problems (Table 1).

**Table 1:** Selected sociodemographic and antenatal characteristics of women included in intervention study.

Characteristics		Denmark (n=54)	Spain (n=50)	Total (N=104)	*P* value	
Gestational age at inclusion (weeks), median (IQR)		22 (4)	10 (2.6)	—^a^	—	
Age (years), mean (SD)		28.7 (5.1)	31.6 (5.9)	30.1 (5.7)	.008^b^	
**Age groups (years), n (%)**						.06^c^
	20-29	31 (57)	16 (32)	46 (45)		
	30-39	16 (30)	25 (50)	41 (39)		
	40-49	2 (4)	2 (4)	4 (4)		
	*Missing*	5 (10)	7 (14)	12 (12)		
**In a relationship, n (%)**						.008^c^
	Yes	39 (72)	47 (94)	86 (83)		
	No	10 (19)	3 (6)	13 (12)		
	*Missing*	5 (9)	0 (0)	5 (5)		
**Living with the relationship partner, n (%)**						<.001^c^
	Yes	35 (65)	41 (82)	75 (73)		
	No	5 (9)	9 (18)	14 (13)		
	*Missing*	14 (26)	0 (0)	14 (13)		
**Educational level, n (%)**						.14^c^
	Primary education	5 (9)	8 (16)	13 (12)		
	Secondary education	18 (33)	17 (34)	35 (34)		
	Higher education/university	26 (48)	25 (50)	51 (49)		
	*Missing*	5 (9)	0 (0)	5 (5)		
**Employment status, n (%)**						<.001^c^
	Employed	29 (54)	32 (64)	61 (59)		
	Unemployed	10 (19)	18 (37)	28 (27)		
	Other: Stay-at-home parent	2 (4)	—^d^	2 (2)		
	Student	8 (14)	—^d^	8 (8)		
	*Missing*	5 (9)	0 (0)	5 (5)		
**Number of children prior to this pregnancy**						.05^b^
	0	25 (46)	19 (38)	44 (42)		
	1	16 (30)	23 (46)	39 (37)		
	2	4 (7)	7 (14)	11 (11)		
	≥3	4 (7)	1 (2)	5 (5)		
	*Missing*	5 (9)	0 (0)	5 (5)		
**Social network, n (%)**						<.001^b^
	Have a person to trust when I have problems	40 (74)	41 (82)	81 (78)		
	Do not have a person to trust when I have problems	1 (20)	9 (18)	10 (10)		
	*Missing*	13 (24)	0 (0)	13 (12)		

^a^Not applicable.

^b^*t* test.

^c^Fisher exact test.

^d^This category was not used in Spain.

## Discussion

### Principal Findings

#### Overview

This paper describes the overall profile of the STOP study and outlines in detail the implementation of digital screening for IPV in antenatal care in Denmark and Spain, along with a digital supportive intervention targeting pregnant women exposed to IPV. We found that digital screening for IPV among pregnant women as part of antennal care is feasible, as 82.65% of the women who were offered the digital screening completed it. However, a digital supportive intervention targeting pregnant women who screen positive for IPV is less feasible, as only 21.4% of the women who were eligible for the intervention received it. Future publications from the STOP study will outline the effectiveness of the intervention in relation to the severity of IPV, postpartum depression, empowerment, and safety actions, as well as in-depth qualitative data on acceptability and feasibility of the intervention.

#### Screening Tool

In both countries, there was a high response rate on the IPV-screening question. The IPV prevalence was indicated by a positive answer to either the AAS or WAST or both; however, the prevalence differed in the two settings, with more women screened as IPV-positive in Spain (17.03%) than in Denmark (8.53%). In Denmark, the screening for IPV was incorporated into the PRO questionnaire, and the responses were added to the women’s electronic health record. In Spain, the IPV screening was incorporated in a separate project app. None of the Spanish midwives had access to the women’s survey answers; only the IPV counselors could access this information. Thus, it seems plausible that more women in Denmark underreported their IPV exposure to avoid their IPV status becoming a permanent part of their medical record, as they may have feared what this could entail for themselves and their babies. If this is the case, and screening for IPV is to be implemented as part of standard antenatal care, national guidelines should be developed as to how midwives should handle both underreporting and exposure to IPV. Further, such guidelines should be communicated to pregnant women prior to screening.

When comparing our findings to the existing literature, our results are somewhat in line with what other studies have previously found. A Cochrane review from 2015 on IPV screening within health care found that different types of screening strategies in antenatal care were feasible and increased disclosure rates of IPV significantly, yet only a few studies used a digital screening approach [21]. Other studies have also demonstrated that digital screening for IPV is feasible in antenatal care [18,37,38]. However, in these studies, digital screening encompassed different digital elements. As digital screening strategies are heterogenous, it is difficult to compare findings and to better understand the feasibility of digital screening within antenatal care compared to, for example, face-to-face screening. We recommend that future studies clearly distinguish between their digital elements.

#### Video Counseling

Despite the intervention being cocreated with key stakeholders, only few women agreed to receive the intervention, with more Spanish women agreeing than Danish women (45.8% and 18.1%, respectively). Part of the reason for this difference in numbers is due to stricter inclusion criteria for the intervention in Denmark. Initially, Danish women were only eligible if they reported severe violence/tensions with a partner on the WAST or exposure to violence within the past 12 months according to the AAS, whereas in Spain, women were also eligible if they reported “some tension” or “some difficulty” with their partner (according to the WAST) or violence at an earlier stage than 12 months (according to the AAS). Eleven months into the study, Denmark changed its AAS criteria to also include women who had been exposed to violence at an earlier stage than 12 months, but not on the WAST criteria. The initial inclusion criteria in Denmark were an attempt to reach only women exposed to violence during pregnancy, but these inclusion criteria may have had the opposite effect and excluded some of the women who deliberately underreported their IPV exposure.

To the best of our knowledge, few studies have evaluated a digital intervention consisting of live counseling sessions for IPV-exposed pregnant women. Hence, it is difficult to compare the feasibility of our intervention. Zlotnik et al [20] tested an advanced avatar-based computer intervention followed up by a phone session with an interventionist; they found a feasibility rate of 17%, which is comparable to the feasibility found in our study. A recent Norwegian RCT explored the effect of a brief video intervention to pregnant women exposed to IPV [39], reporting a higher feasibility rate of 33%. The current literature is ambiguous, and the topic should be investigated further before making recommendations about the feasibility of providing pregnant women with a digital supportive intervention. However, it is plausible that pregnancy may not be the ideal timing for an intervention among pregnant women who screen positive for IPV.

### Strengths and Limitations

Our study has several strengths and limitations. A major strength of the STOP study is that this is the first large-scale cross-country study in Europe that has implemented both digital screening and a digital intervention to support pregnant women exposed to IPV. Hereby, this study provides both country-specific and cross-cultural evidence of the potential and challenges for digital solutions within the field of IPV in antenatal care. Another key strength is that data were collected prospectively, which limits the risk of information bias. Further, both the screening and intervention were implemented in large nonselected populations in both Denmark and Spain, which heightens the generalizability of our results. Additionally, the intervention was cocreated with women who had previously been exposed to IPV, psychologists, midwives, and NGOs working within the field of IPV, which ensured that the content and structure of the intervention were in line with the needs of the target group.

A limitation of our study is that there is no “gold standard” for how to measure exposure to IPV. For this reason, the Danish and Spanish teams used two different validated screening tools and had different inclusion criteria for the severity of the violence regarding whether women were eligible for the intervention. Further, neither of the tools we used differentiates between different forms of emotional violence such as stalking or digital violence. This could imply that certain types of emotional violence were not identified during the screening. Hence, there is a need for further development of screening tools for IPV and agreeing upon a gold standard on how to measure exposure to IPV.

Another limitation of our study is that women were only screened digitally for IPV once, namely at the beginning of their pregnancy. However, in the Danish setting, midwives followed up on the digital screening during the first antenatal appointment, although this was not done systematically for all women who screened negative for IPV. The rescreening resulted in a positive IPV screening result among five women who had initially not responded to the digital screening and six women who initially screened IPV-negative. The reason for this may be that pregnant women need time to realize that they are exposed to IPV or need more time to feel “ready” to disclose and reach out for help.

Hence, we recommend for future studies, or if the intervention is to be implemented in clinical practice, that the digital screening is repeated in the second and/or third trimester and that missing screening results are systematically followed up during face-to-face antenatal care appointments. Finally, our study excluded non-Danish– or non-Spanish–speaking women, as the setup did not allow for the involvement of interpreters. This limits the generalization of our results to immigrant populations who speak neither Danish nor Spanish. This group may be particularly vulnerable in relation to IPV, and we recommend that future studies address this issue by training IPV counselors who speak the most common immigrant languages.

### Conclusion

It is highly feasible to digitally screen pregnant women for IPV as part of antenatal care in Denmark and Spain, but it is less feasible to support IPV-exposed pregnant women through a digital intervention during pregnancy. We recommend more research within this field as existing studies are limited. We further recommend that digital screening be repeated throughout pregnancy and followed up face-to-face during antenatal care appointments to allow for more women to obtain the support they need whenever they are ready. Future publications from the STOP cohort will provide data on the prevalence and type of IPV among pregnant women, outline the effect of the intervention on selected health outcomes, and provide in-depth data on the acceptability and feasibility of the digital intervention.

## Abbreviations

AAS: Abuse Assessment Screen
EPDS: Edinburgh Postnatal Depression Scale
IPV: intimate partner violence
ISA: Index of Spouse Abuse
MAST: Model for Assessment of Telemedicine
MOVERS: Measure of Victim Empowerment Related to Safety
NGO: nongovernmental organization
PRO: patient-reported outcome
RCT: randomized controlled trial
REDCap: Research Electronic Data Capture
STOP: Stop Intimate Partner Violence in Pregnancy
WAST: Women Abuse Screening Tool

## Data Availability

Due to Danish legislation, the data set generated from the project cannot immediately be shared without specific consent from the national Danish data management administration.
